# The Effectiveness of Liraglutide in Nonalcoholic Fatty Liver Disease Patients with Type 2 Diabetes Mellitus Compared to Sitagliptin and Pioglitazone

**DOI:** 10.1100/2012/496453

**Published:** 2012-08-13

**Authors:** Takamasa Ohki, Akihiro Isogawa, Masahiko Iwamoto, Mitsuru Ohsugi, Haruhiko Yoshida, Nobuo Toda, Kazumi Tagawa, Masao Omata, Kazuhiko Koike

**Affiliations:** ^1^Department of Gastroenterology, Mitsui Memorial Hospital, Kanda-izumicho 1, Chiyoda-ku, Tokyo 101-8643, Japan; ^2^Department of Diabetes and Metabolism, Mitsui Memorial Hospital, Kanda-izumicho 1, Chiyoda-ku, Tokyo 101-8643, Japan; ^3^Department of Gastroenterology, University of Tokyo, 7-3-1 Hongo, Bunkyo-ku, Tokyo 113-8655, Japan; ^4^Yamanashi Prefectural Hospital Organization, 1-1-1 Fujimi, Kofu City 400-8506, Japan

## Abstract

*Background*. Liraglutide leading to improve not only glycaemic control but also liver inflammation in non-alcoholic fatty liver disease (NAFLD) patients. *Aims*. The aim of this study is to elucidate the effectiveness of liraglutide in NAFLD patients with type 2 diabetes mellitus (T2DM) compared to sitagliptin and pioglitazone. *Methods*. We retrospectively enrolled 82 Japanese NAFLD patients with T2DM and divided into three groups (liraglutide: *N* = 26, sitagliptin; *N* = 36, pioglitazone; *N* = 20). We compared the baseline characteristics, changes of laboratory data and body weight. *Results*. At the end of follow-up, ALT, fast blood glucose, and HbA1c level significantly improved among the three groups. AST to platelet ratio significantly decreased in liraglutide group and pioglitazone group. The body weight significantly decreased in liraglutide group (81.8 kg to 78.0 kg, *P* < 0.01). On the other hands, the body weight significantly increased in pioglitazone group and did not change in sitagliptin group. Multivariate regression analysis indicated that administration of liraglutide as an independent factor of body weight reduction for more than 5% (OR 9.04; 95% CI 1.12–73.1, *P* = 0.04). *Conclusions*. Administration of liraglutide improved T2DM but also improvement of liver inflammation, alteration of liver fibrosis, and reduction of body weight.

## 1. Introduction

Nonalcoholic fatty liver disease (NAFLD) is reported to be the most common liverdisease, increasing in prevalence in Western countries as well as in Japan because of the raising prevalence of obesity [1, 2]. NAFLD shows a wide disease spectrum ranging from simple steatosis to steatohepatitis and finally to cirrhosis. Approximately 3% of the patients who have NAFLD will develop cirrhosis [3]. The main pathophysiological problem in NAFLD patients is insulin resistance. Thus, there is a clear association between NAFLD and metabolic syndrome which induces type 2 diabetes mellitus (DM), obesity, hypertension, and dyslipidemia [4]. Improvement of insulin resistance and sensitivity has therapeutic effect in preventing the progression of NAFLD because the accumulation of triglycerides in hepatocytes is considered to be the first step in the current two-hit theory of the pathophysiological development of NAFLD [5]. Several studies indicated that improving insulin resistance and sensitivity would reduce fatty liver change and might prevent the second step of hepatocytes injury due to oxidative stress [6–8].

Glucagon like peptide-1 (GLP-1) is a naturally existing incretin hormone with a potent blood-glucose reducing action only during hyperglycemia because it induces insulin secretion and reduces glucagon secretion in a glucose-dependent mechanism [9]. In addition, GLP-1 prolongs gastric emptying and induces satiety, leading to decreased energy intake and body weight [10, 11]. Therefore, GLP-1 has a great potential among type 2 DM patients. However, its half-life is extremely short because GLP-1 is rapidly degraded by the enzyme dipeptidyl peptidase-4 (DPP-4) [9]. Liraglutide, one of the GLP-1 analogues, has 97% aminoacid sequence identity to native human GLP-1 and an acyl side-chain attachment, which makes it bind to albumin. These small structural differences prolong the half-life of GLP-1 to 13 hours, making it possible for once daily administration [12].   Several studies showed that liraglutide was well tolerated, improved glycaemic control with a low risk of hypoglycemia, improved functions of beta-cell, and was associated with body weight reduction [13]. The receptors of GLP-1 analogue also exist in human hepatocytes and administration of GLP-1 analogue reported to directly reduce liver steatosis and fibrosis in *in vivo* study [14, 15].

DPP-4 inhibitors (DPP-4I) are also novel drugs as GLP-1 analogue which affect incretin hormone. DPP-4 is one of the serine proteases enzymes that lead inactivation of incretin hormone such as GLP-1. DPP-4I is a class of oral hypoglycemics that block the activity of DPP-4. The mechanism of DPP-4I is to increase GLP-1 levels, which inhibit glucagon release, which in turn increases insulin secretion, decreases gastric emptying, and decreases blood glucose levels [16]. Serum DPP-4 activity is reported to be significantly higher in NAFLD patients [17]. Thus, administration of DPP-4I might have possibility to improve fatty liver change as same mechanism as GLP-1 analogue. However, the effectiveness of DPP-4 inhibitors on NAFLD patients is still unknown. Future use of GLP-1 analogue and DPP-4I for NAFLD may be significant advance in treatment of this common form of disease.

On the other hand, pioglitazone has already several clinical evidences on treatment of NAFLD [18]. Pioglitazone, a thiazolidinedione derivative (TZD), is a peroxisome proliferator-activated receptor *γ* (PPAR*γ*) agonist that ameliorates insulin resistance and improves glucose and lipid metabolism in type 2 DM [19]. Insulin resistance in NAFLD is frequently associated with chronic hyperinsulinemia, hyperglycemia, and an excessive supply of plasma free fatty acids to the liver. Pioglitazone reverses these abnormalities by improving insulin resistance in adipose tissues, the liver, and muscles [20]. However, there is a disadvantage of increasing body weight [21] which may affect on long-term outcomes because weight reduction is one of the important treatment of NAFLD [8]. According to these backgrounds, we conducted this retrospective cohort study to compare the efficacy and effectiveness among liraglutide, one of the GLP-1 analogues compared to sitagliptin, one of the DDP-4 inhibitors and pioglitazone.

## 2. Patients and Methods

### 2.1. Patients

**Figure 1:**
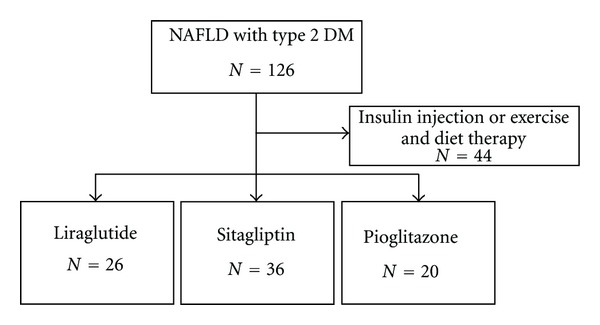
A total of 126 patients who were clinically diagnosed NAFLD with type 2 DM visited the outpatient clinic of Department of Diabetes and Metabolism or Department of Gastroenterology, Mitsui Memorial Hospital. We retrospectively analyzed 82 of them, excluding 44 patients only treated with insulin injection or exercise and diet therapy. We divided the rest 82 patients into three groups: liraglutide-treated group (*N* = 26), sitagliptin-treated group (*N* = 36), and pioglitazone-treated group (*N* = 20).

 Between April 1, 2003 and March 31, 2011, a total of 126 patients who were clinically diagnosed NAFLD with type 2 DM visited the out patient clinic of Department of Diabetes and Metabolism or Department of Gastroenterology, Mitsui Memorial Hospital. We retrospectively analyzed 82 of them, excluding 44 patients only treated with insulin injection or exercise and diet therapy. We divided the rest 82 patients into three groups: liraglutide-treated group (*N* = 26), sitagliptin-treated group (*N* = 36), and pioglitazone-treated group (*N* = 20) (Figure 1). All of these patients were negative for hepatitis B and C virus infection, anti-mitochondrial antibody, and anti-nuclear antibody. Hemochromatosis and Wilson's disease were diagnosed in none of them. Clinical diagnosis of NAFLD was based on the following criteria: existence of fatty liver change in ultrasonography, alcohol consumption less than 20 g ethanol per day, and continuous elevation of alanine aminotransferase (ALT) equal or over 40 IU/L for more than 6 months. Diagnosis of DM was based on medical history or 75 g oral glucose tolerance test. Dyslipidemia was defined as blood total cholesterol concentration over 220 mg/dL or triglyceride over 150 mg/dL, or history of taking oral drugs for dyslipidemia. Hypertension was defined as systolic blood pressure over 140 mmHg or diastolic blood pressure over 90 mmHg, or taking oral drugs for hypertension. Body mass index (BMI) was calculated as body weight in kilogram (kg) divided twice by body height in meter (m), which was also routinely measured at the beginning of the treatment. The evaluation of liver fibrosis depended on calculation of aspartate aminotransferase (AST) to platelet counts ratio (APRI) index [22]. APRI index was calculated as AST level (IU/L) divided by upper limit of AST (37 IU/L) and platelet counts (×10^9^/L), and finally multiplied by 10^2^. APRI over 1.5 was considered as bridging fibrosis and over 2.0 as liver cirrhosis. This study was conducted according to STROBE statement [23].

### 2.2. Treatment and Followup

 All patients were treated in our out patient clinic and had uncontrollable type 2 diabetes (HbA1c over 6.5%) with exercise and diet therapy. The administration of each medicine, liraglutide or sitagliptin or pioglitazone, was determined by our out patient clinic doctors. Liraglutide was subcutaneously injected once daily 0.3 mg for the first week, 0.6 mg for the next week, and finally up to the limit dose 0.9 mg if necessary. Sitagliptin was administered via oral route once daily 50 mg up to 100 mg if necessary. Pioglitazone was administered once daily 15 mg via oral route.

The beginning of followup was defined as the administration date of each medicine, and the end of follow-up was September 30, 2011. The followup consisted of monthly or bimonthly physical examination including body weight measurement and blood tests. Patients who completely changed each medicine to intensive therapies such as insulin injection because of exacerbation of diabetes were treated as end of follow at the moment of treatment change. Even if other oral glucose-lowering agents were added, the followup was considered to be valid as long as each medicine had still been continued. Patients who quitted each treatment due to improvement of diabetes were also treated as end of follow at the moment. The final decision of exacerbation or improvement of diabetes was made by our out patient clinic doctors' own assessments.

### 2.3. Statistical Analyses

 Data were expressed as the median and range (25th–75th percentiles) unless otherwise indicated. Continuous variables among the three groups were compared by analysis of variance (ANOVA). Categorical variables were compared by chi-square test. Changes of parameters after the administration of each medicine were compared by paired *t*-test. There was no missing data. As previously mentioned, body weight reduction is an important treatment method for NAFLD patients [8]. Thus, we also tested the following variables obtained at the time of entry in univariate and multivariate logistic regression analysis to evaluate the factors which contribute to body weight reduction for over 5%; age, sex, BMI, presence of hypertension, existence of dyslipidemia, history of smoking, combination use of metformin or sulfonylurea agents, AST, ALT, *γ*-glutamyl transpeptidase (*γ*-GTP), fast blood sugar level, HbA1c, LDL-cholesterol, triglyceride, platelet counts, APRI index, and treatment modalities (liraglutide, sitagliptin, or pioglitazone). Parameters which *P* values were less than 0.10 included in the multivariate analysis. Nominal categorical data were represented by corresponding binary dummy variables. Data processing and analysis were performed using the StatView version 5.0 (SAS Institute Inc.).

## 3. Results

### 3.1. Patient Profile

 The patients were divided into three groups according to the treatment modalities: liraglutide-treated group (*N* = 26), sitagliptin-treated group (*N* = 36), and pioglitazone-treated group (*N* = 20) (Figure 1). Baseline characteristics of each group were shown in Table 1. Dosing period of each drudges significantly different among the three groups (*P* < 0.01). The longest treatment period was observed in pioglitazone group. There were significant differences about the comorbidity with dyslipidemia among the three groups (*P* < 0.01). Comorbidity with dyslipidemia was higher in liraglutide group (92.3%). The combination use of metformin or sulfonylurea agents was significantly different among the three groups (*P* < 0.01 and *P* = 0.04, resp.). The proportion of combination use with metformin agents was higher in sitagliptin group (30.6%), and the proportion of combination use with sulfonylurea agents was higher in liraglutide group (69.2%). There were also significant differences about AST level, LDL-cholesterol level, and APRI index among the three groups (*P* = 0.04, *P* = 0.02, and *P* = 0.04, resp.). Pioglitazone group had advanced liver inflammation and fibrosis. There were no significant differences about other baseline characteristics among the three groups: age, proportion of male patients, body weight, BMI, comorbidity with hypertension, proportion of smoking patients, ALT level, *γ*-GTP level, fast blood glucose level, HbA1c level, Triglyceride level, and platelet counts.

### 3.2. Changes of Parameters after the Administration of Each Treatment

Table 2 showed the change of each parameter after administration of liraglutide. There was significant decrement in body weight and BMI; body weight decreased from 81.8 kg to 78.0 kg, and BMI decreased and from 30.1 kg/m^2^ to 28.6 kg/m^2^ (both *P* < 0.01). The control of diabetes mellitus also markedly improved: fast blood glucose decreased from 207 mg/dL to 168 mg/dL (*P* = 0.02) and HbA1c decreased from 8.4% to 7.6% (*P* = 0.01). Additionally, there was significant improvement of liver inflammation and liver fibrosis score; AST decreased from 50 IU/L to 35 IU/L, ALT decreased from 65 IU/L to 48 IU/L, and APRI index decreased from 0.73 to 0.49 (all *P* < 0.01).

Table 3 showed the change of each parameter after administration of sitagliptin. The control of diabetes mellitus significantly improved: fast blood glucose decreased from 175 mg/dL to 152 mg/dL and HbA1c decreased from 8.4% to 7.3% (both *P* = 0.01). LDL-cholesterol also markedly decreased from 126 mg/dL to 113 mg/dL (*P* = 0.02). Additionally, there was significant improvement of liver inflammation; ALT decreased from 75 IU/L to 61 IU/L (*P* = 0.03), and *γ*-GTP decreased from 89 IU/L to 75 IU/L (*P* = 0.01). However, body weight, BMI, AST level, and APRI index changes did not retain statistical significance.

Table 4 showed the change of each parameter after administration of pioglitazone. The control of diabetes mellitus significantly improved: fast blood glucose decreased from 182 mg/dL to 141 mg/dL, and HbA1c decreased from 7.7% to 6.9% (both *P* = 0.01). Triglyceride also markedly decreased from 210 mg/dL to 161 mg/dL (*P* = 0.03). Additionally, there was significant improvement of liver inflammation and liver fibrosis score; AST decreased from 62 IU/L to 41 IU/L, ALT decreased from 87 IU/L to 53 IU/L, *γ*-GTP decreased from 95 IU/L to 65 IU/L, and APRI index decreased from 0.96 to 0.73 (all *P* < 0.01). However, there was significant increment in body weight and BMI; body weight increased from 78.6 kg to 81.8 kg (*P* < 0.01), and BMI increased 28.8 kg/m^2^ to 30.0 kg/m^2^ (*P* = 0.02).

### 3.3. Logistic Regression Analysis about 5% Body Weight Reduction

 As previously mentioned, body weight reduction for more than 5% is one of the important treatment of NAFLD [8]. Thus, we performed univariate and multivariate logistic regression analysis to clarify the parameters which affect on body weight reduction for over 5% (Table 5). In the univariate logistic regression analysis, administration of liraglutide, higher fast blood glucose level, and higher APRI index score were identified as significant factors contributing to body weight reduction (*P* < 0.01, *P* < 0.01, and *P* = 0.04, resp.). Combination use of sulfonylurea agents and higher AST level had also tendency to be significant factors affecting on body weight loss (*P* = 0.07 and *P* = 0.09, resp.). On the other hand, administration of sitagliptin was identified as a significant adverse factor on body weight reduction (*P* < 0.01). Higher serum albumin level also tended to be an adverse factor on body weight loss (*P* = 0.06). Adjusting for these factors, multivariate logistic regression analysis indicated that administration of liraglutide and higher fast blood glucose level as independent factors affecting on body weight reduction for over 5% (both *P* = 0.04).

## 4. Discussion

 NAFLD is a liver disease that is characterized histologically by hepatic steatosis, lobular inflammation, and hepatocellular ballooning [24], and it was reported that at least 3% of the patients progressed to cirrhosis [3]. The disorder is thought to be common because the incidence of its typical features, fatty liver disease, obesity, and type 2 DM, is increasing [25]. Multiple pharmacologic interventions have been attempted with variable success. Particularly, trials of glucose lowering agents such as metformin and pioglitazone have yielded promising results [18, 26]. In this study, drastic improvement of serum AST and ALT level was shown not only in pioglitazone group but also in liraglutide group and sitagliptin group, suggesting that treatment of diabetes improved insulin resistance and led to amelioration of liver inflammation in NAFLD patients with type 2 DM.

 However, the improvement of liver inflammation and liver fibrosis is different matter. Since in a randomized controlled trial intended for NAFLD patients, pioglitazone demonstrated alteration of liver inflammation but did not affect on improvement of liver fibrosis [6]. Since this current study was based on outpatient clinic medial care, liver biopsy was not applied in our study population. Noninvasive measurement methods of liver stiffness, such as transient elastography, were not available in our institute. Thus, we applied APRI index to evaluate the degree of liver fibrosis [22]. In this current study, APRI index significantly improved between liraglutide group and pioglitazone group but not in sitagliptin group. The calculation of APRI index depended on changes in AST level and it might be one of the limitations of this study. Nonetheless, the alteration of APRI index might be expected in these two groups. On the other hand, sitagliptin group had already lower serum AST level at the time of administration. It might be the main cause of no change in the APRI index at the end of followup. Although we could not clarify the true outcome of liraglutide on liver fibrosis, it was reported that GLP-1 analogue directly inhibited fibroblast growth factor 21 which promoted the progression of liver fibrosis in mice model [15]. Further more studies are needed.

 Obesity is considered one of the most important risk factors for NAFLD [27]. Weight reduction via lifestyle intervention is generally recommended as an initial step in the management of NAFLD [27], and its effectiveness was proven in a randomized controlled trial [8]. However, lifestyle intervention depends on patient's own efforts and sometimes difficult to achieve the aim [28]. In this current study, the body weight dramatically changed after the administration of each treatment. Significant body weight reduction was shown in liraglutide group, significant body weight gain was shown in pioglitazone group, and body weight did not change in sitagliptin group. These results were supported by previous reports [13]. Besides, multivariate logistic regression analysis indicated that administration of liraglutide as an independent factor for body weight reduction. Although the first step in the management of NAFLD is lifestyle intervention, liraglutide may support body weight reduction via suppressing appetite and finally affect on improvement of NAFLD.

 Since this current study based on a retrospective cohort, there were lots of limitations. The first limitation is that the difference of dosing period of each medicine. The median dosing period of pioglitazone was about 1200 days. On the other hand, the median dosing period of liraglutide and sitagliptin was about 340 days and 250 days, respectively. There was approximately fourfold difference, and the long-term outcome of liraglutide and sitagliptin was still unknown. The second limitation is that liraglutide is administered by subcutaneous injection. Sitagliptin and pioglitazone are oral drugs and considered to be less invasive than liraglutide. Continuing subcutaneous injection every day may be a great stress for patients even if weekly subcutaneous injective GLP-1 analogue (exenatide long-acting release) will be available in the near future. The last limitation is that this study cohort was consisted in patients treated with combination use of metformin agent. Metformin agent was reported to have some effects on liver steatosis and inflammation [26].

 In conclusion, we have demonstrated the improvement of liver inflammation and diabetes in NAFLD patients with type 2 DM treated by liraglutide, sitagliptin, and pioglitazone. However, APRI index did not alter in sitagliptin group, and body weight significantly increased in pioglitazone group. Aggravation of liver fibrosis score might lead future liver cirrhosis, and body weight gain could exacerbate liver inflammation and other metabolic disorders. Administration of liraglutide led not only to good control of type 2 DM but also improvement of liver inflammation, alteration of liver fibrosis, and reduction of body weight. Particularly, body weight reduction was a favorable outcome of applying liraglutide in NAFLD patients with type 2 DM.

## Figures and Tables

**Table 1:** Baseline characteristics of patients.

Variable	Total	GLP-1 group	DPP-4 group	Pioglitazone group	*P*
	(*n* = 82)	(*n* = 26)	(*n* = 36)	(*n* = 20)	
Mean follow-up period (days)	520 (222–410)	342 (291–392)	250 (187–233)	1236 (378–2216)	<0.01^‡^
Age^∗^ (years old)	54.2 (44.4–63.2)	55.7 (50.2–62.3)	54.0 (42.7–64.3)	52.7 (46.3–59.6)	0.71^†^
Male, *n* (%)	61 (74.4%)	18 (69.2%)	29 (80.6%)	14 (70.0%)	0.55^‡^
Body weight^∗^ (kg)	80.7 (69.1–89.7)	81.8 (74.4–92.3)	81.1 (68.4–88.9)	78.6 (68.2–86.0)	0.79^†^
BMI^∗^ (kg/m^2^)	29.4 (25.7–31.7)	30.1 (26.8–32.1)	29.4 (25.1–31.3)	28.8 (24.6–31.5)	0.75^†^
Hypertension, *n* (%)	41 (50.0%)	17 (65.4%)	16 (44.4%)	8 (40.0%)	0.17^‡^
Dyslipidemia, *n* (%)	57 (69.5%)	24 (92.3)	19 (52.8%)	14 (70.0%)	<0.01^‡^
Smoking, *n* (%)	36 (43.9%)	11 (42.3%)	16 (44.4%)	9 (45.0%)	0.97^‡^
Sulfonylurea agent, *n* (%)	40 (48.8%)	18 (69.2%)	15 (41.7%)	7 (35.0%)	0.04^‡^
Metformin agent, *n* (%)	14 (17.1%)	0 (0%)	11 (30.6%)	3 (15.0%)	<0.01^‡^
AST^∗^ (IU/mL)	52 (35–61)	50 (32–59)	47 (36–56)	62 (40–85)	0.04^†^
ALT^∗^ (IU/mL)	75 (55–92)	65 (52–74)	75 (55–89)	87 (60–112)	0.07^†^
*γ*-GTP^∗^ (IU/mL)	93 (49–120)	98 (55–127)	89 (47–116)	95 (54–114)	0.85^†^
Fast blood glucose^∗^ (mg/dL)	187 (139–229)	207 (151–256)	175 (138–201)	182 (135–224)	0.10^†^
HbA1c (%)	8.2 (7.2–9.3)	8.4 (7.4–9.4)	8.4 (7.5–9.5)	7.7 (6.9–8.5)	0.22^†^
LDL-cholesterol^∗^ (mg/dL)	115 (95–140)	103 (78–116)	126 (104–148)	114 (92–140)	0.02^†^
Triglyceride^∗^ (mg/dL)	191 (117–240)	199 (125–283)	175 (114–229)	210 (130–198)	0.59^†^
Platelet count^∗^ (×10^3^/*μ*L)	206 (167–240)	204 (183–230)	216 (166 –248)	192 (147–233)	0.38^†^
APRI index^∗^	0.75 (0.40–0.95)	0.73 (0.37–0.91)	0.64 (0.44–0.76)	0.96 (0.60–1.09)	0.04^†^

^∗^Expressed as median (25th–75th percentiles).

^†^ANOVA.

^‡^Chi-square tests.

BMI: body mass index; AST: aspartate aminotransferase; ALT: alanine aminotransferase; *γ*-GTP: *γ*-glutamyl transpeptidase; HbA1c: hemoglobin A1c; APRI: AST-to-platelet counts ratio index.

**Table 2:** Change of parameters after administration of liraglutide.

	Liraglutide group (*N* = 26)		
Variables (*N*)	Before administration	After administration	Paired *t*-test
			*P* value
Body weight^∗^ (kg)	81.8 (74.4–92.3)	78.0 (72.1–88.5)	<0.01
BMI^∗^ (kg/m^2^)	30.1 (26.8–32.1)	28.6 (25.5–31.6)	<0.01
AST^∗^ (IU/mL)	50 (32–59)	35 (29–39)	<0.01
ALT^∗^ (IU/mL)	65 (52–74)	48 (34–61)	<0.01
*γ*-GTP^∗^ (IU/mL)	98 (55–127)	90 (48–130)	0.44
Fast blood glucose^∗^ (mg/dL)	207 (151–256)	168 (116–199)	0.02
HbA1c (%)	8.4 (7.4–9.4)	7.6 (6.9–8.4)	0.01
LDL-cholesterol^∗^ (mg/dL)	103 (78–116)	99 (78–115)	0.51
Triglyceride^∗^ (mg/dL)	199 (125–283)	175 (114–231)	0.19
Platelet count^∗^ (×10^3^/*μ*L)	204 (183–230)	207 (173–224)	0.67
APRI index^∗^	0.73 (0.37–0.91)	0.49 (0.34–0.56)	<0.01

BMI: body mass index; AST: aspartate aminotransferase; ALT: alanine aminotransferase; *γ*-GTP: *γ*-glutamyl transpeptidase; HbA1c: hemoglobin A1c; APRI: AST-to-platelet counts ratio index.

**Table 3:** Change of parameters after administration of sitagliptin.

	Sitagliptin (*N* = 36)		
Variables (*N*)	Before administration	After administration	Paired *t*-test
			*P* value
Body weight^∗^ (kg)	81.1 (68.4–88.9)	80.7 (68.7–86.3)	0.39
BMI^∗^ (kg/m^2^)	29.4 (25.0–31.3)	29.2 (25.5–31.2)	0.56
AST^∗^ (IU/mL)	47 (36–56)	44 (30–45)	0.47
ALT^∗^ (IU/mL)	75 (55–89)	61 (40–74)	0.03
*γ*-GTP^∗^ (IU/mL)	89 (47–116)	75 (43–100)	0.01
Fast blood glucose^∗^ (mg/dL)	175 (138–201)	152 (128–187)	<0.01
HbA1c (%)	8.4 (7.5–9.5)	7.3 (6.5–7.8)	<0.01
LDL-cholesterol^∗^ (mg/dL)	126 (104–148)	113 (97–129)	0.02
Triglyceride^∗^ (mg/dL)	175 (114–229)	166 (99–195)	0.60
Platelet count^∗^ (×10^3^/*μ*L)	216 (166–248)	202 (162–240)	<0.01
APRI index^∗^	0.64 (0.44–0.76)	0.60 (0.39–0.63)	0.47

BMI: body mass index; AST: aspartate aminotransferase; ALT: alanine aminotransferase; *γ*-GTP: *γ*-glutamyl transpeptidase; HbA1c: hemoglobin A1c; APRI: AST-to-platelet counts ratio index.

**Table 4:** Change of parameters after administration of pioglitazone.

	Pioglitazone group (*N* = 20)		
Variables (*N*)	Before administration	After administration	Paired *t*-test
			*P* value
Body weight^∗^ (kg)	78.6 (68.2–86.0)	81.8 (73.0–86.8)	<0.01
BMI^∗^ (kg/m^2^)	28.8 (24.6–31.5)	30.0 (26.2–33.9)	0.02
AST^∗^ (IU/mL)	62 (40–85)	41 (26–47)	<0.01
ALT^∗^ (IU/mL)	87 (60–112)	53 (33–69)	<0.01
*γ*-GTP^∗^ (IU/mL)	95 (54–114)	65 (31–87)	<0.01
Fast blood glucose^∗^ (mg/dL)	182 (135–224)	141 (114–161)	<0.01
HbA1c (%)	7.7 (6.9–8.5)	6.9 (6.2–7.3)	<0.01
LDL-cholesterol^∗^ (mg/dL)	114 (92–140)	114 (87–140)	0.78
Triglyceride^∗^ (mg/dL)	210 (130–198)	161 (95–165)	0.03
Platelet count^∗^ (×10^3^/*μ*L)	193 (147–233)	184 (138–226)	0.14
APRI index^∗^	0.96 (0.60–1.09)	0.73 (0.41–0.71)	0.01

BMI: body mass index; AST: aspartate aminotransferase; ALT: alanine aminotransferase; *γ*-GTP: *γ*-glutamyl transpeptidase; HbA1c: hemoglobin A1c; APRI: AST-to-platelet counts ratio index.

**Table 5:** Univariate and multivariate logistic regression analysis to evaluate the factors which contribute to body weight reduction for over 5%.

Variables	Odds ratio (95% CI) univariate	*P*	Odds ratio (95% CI) multivariate	*P*
Age (per year)	1.02 (0.97–1.07)	0.54		
Male	1.32 (0.33–5.28)	0.69		
Body weight (per 1.0 kg)	1.01 (0.98–1.05)	0.49		
BMI (per 1.0 kg/m^2^)	1.06 (0.96–1.17)	0.23		
Hypertension	1.41 (0.44–4.51)	0.56		
Dyslipidemia	1.12 (0.31–3.97)	0.86		
Smoking	1.91 (0.59–6.10)	0.28		
Liraglutide	8.13 (2.24–29.5)	<0.01	9.04 (1.12–73.1)	0.04
Sitagliptin	0.17 (0.03–0.80)	<0.01	1.19 (0.12–12.0)	0.88
Pioglitazone	0.46 (0.09–2.27)	0.34		
Metformin	2.66 (0.08–9.30)	0.96		
Sulfonylurea	3.17 (0.90–11.1)	0.07	1.61 (0.28–9.43)	0.60
Albumin (per 1.0 mg/dL)	0.13 (0.01–1.13)	0.06	0.11 (0.01–2.42)	0.16
AST (per 10 IU/L)	1.22 (0.97–1.61)	0.09	1.03 (0.95–1.11)	0.47
ALT (per 10 IU/L)	1.05 (0.88–1.25)	0.54		
*γ*-GTP (per 10 IU/L)	1.05 (0.94–1.15)	0.33		
Fast blood glucose (per 10 mg/dL)	1.13 (1.03–1.26)	<0.01	1.14 (1.01–1.28)	0.04
HbA1c (per 1.0%)	1.32 (0.85–2.02)	0.21		
LDL-cholesterol (per 10 mg/dL)	0.87 (0.71–1.06)	0.16		
Triglyceride (per 10 mg/dL)	1.02 (0.98–1.06)	0.41		
Platelet counts (per ×10^4^/*μ*L)	0.91 (0.82–1.02)	0.09	0.98 (0.72–1.33)	0.42
APRI index (per 1.0)	3.22 (0.01–997)	0.04	2.11 (0.01–332)	0.77

CI: confidence interval; BMI: body mass index; AST: aspartate aminotransferase; ALT: alanine aminotransferase; *γ*-GTP: *γ*-glutamyl transpeptidase; HbA1c: hemoglobin A1c; APRI: AST-to-platelet counts ratio index.

## Abbreviations

GLP-1: Glucagon like peptide-1
NAFLD: Nonalcoholic steatohepatitis
DM: Diabetes mellitus
DPP-4I: Dipeptidyl peptidase-4 inhibitor
DPP-4: Dipeptidyl peptidase-4
TZD: Thiazolidinedione
NASH: Nonalcoholic steatohepatitis
AST: Aspartate aminotransferase
ALT: Alanine aminotransferase
APRI: AST to platelet counts ratio index
HbA1c: Hemoglobin A1c
PPAR*γ*: Peroxisome proliferator-activated receptor *γ*
BMI: Body mass index
ANOVA: Analysis of variance
*γ*-GTP: *γ*-glutamyl transpeptidase.
